# Prevalence and morphological changes of carotid kinking and coiling in growth: an echo-color Doppler study of 2856 subjects between aged 0 to 96 years

**DOI:** 10.1007/s10554-020-02014-0

**Published:** 2020-09-10

**Authors:** Luigi Di Pino, Antonio G. Franchina, Serena Costa, Stella Gangi, Francesco Strano, Mario Ragusa, Luca Costanzo, Corrado Tamburino, Davide Capodanno

**Affiliations:** 1 Cardiology and Angiology, Department of Cardiovascular Disease, C.A.S.T., A.O.U. “Policlinico-Vittorio Emanuele”, University of Catania, Catania, Italy; 2 Angiology Unit, San Marco Hospital, Department of Cardiovascular Disease, A.O.U. “Policlinico-Vittorio Emanuele”, University of Catania, Catania, Italy

**Keywords:** Internal carotid kinking, Carotid artery anomalies, Carotid artery dolichoarteriopathies, Etiopathogenetic hypothesis, Echo-color Doppler, Growth

## Abstract

Extracranial internal carotid artery (EICA) kinking and coiling are the most frequently reported carotid anomalies in the literature. Embryogenic and acquired causes for such anomalies have been postulated but the prevalence of kinking and coiling has not been well characterized across age categories. The aim of this study is to evaluate the prevalence of EICA coiling and kinking among different age groups to better understand its potential causes and changes during the course of life. A total of 2856 subjects aged 0 to 96 years were studied by echo-color Doppler (ECD). Morphology and anatomical anomalies of the EICA were assessed. Patients with anatomical anomalies were stratified by age groups and the prevalence of EICA abnormalities was calculated. The maximal velocity recorded at the level of the kinking was compared with that measured in the common carotid artery and the peak systolic velocity kinking ratio (PSVKR) was calculated. A total of 284 subjects (9.94% of the sample) were found to have kinking or coiling of the EICA. The prevalence was significantly higher at the extremes of age (≤ 20 and > 60 years old, p < 0.001) supporting the hypothesis of a reduction with growth and a new increase in the elderly. PSVKR was higher in subjects with more severity kinking. This study showed a higher prevalence of EICA coiling and kinking in the very young and in the elderly. This bimodal prevalence distribution supports the hypothesis of a congenital anomaly that resolves with somatic growth, while advanced age with its many anatomical changes leads to its reappearance or worsening. Studies with longitudinal follow-up and paired observation are required to support this hypothesis.

## Introduction

Morphological anomalies of the extracranial internal carotid artery (EICA), also named carotid dolichoarteriopathies, are frequent in the general population, ranging between 10% and 45% [1, 2]. The clinical relevance of EICA dolichoarteriopathies stems from their putative link with reduction of cerebral blood flow, especially if atherosclerotic carotid narrowing coexists [3, 4]. Weibel and Fields classified EICA anomalies into three types: *tortuosity* (i.e., elongation and ripple of the EICA with C- or S- shape), *coiling* (i.e., elongation of the EICA resulting in an exaggerated S-shaped curve or circular configuration) and *kinking* (i.e., acute angulation of the EICA) [5].

Compared with tortuosity, coiling and kinking are associated with more hemodynamic abnormalities and flow impairment. Coiling of the carotid artery may produce luminal narrowing leading to turbulent blood flow and symptoms of cerebrovascular insufficiency (strokes, transient ischemic attacks, amaurosis fugax), similar to those caused by atherosclerotic disease. Among children, coiling is often the reason for reduced cognitive capacity, slow neuropsychological development and focal or grand mal convulsions [6, 7]. Kinking is the most frequently reported (5–25%) and clinically relevant type of carotid abnormalities [8]. Kelly et al. postulated two theories for kinking [9], including (1) failure of the embryological developmental process with kink persistence and (2) morphological changes with age resulting in elongation and tortuosity of the artery. The embryogenic hypothesis has been confirmed in some studies [1, 10], but not in others that link this anatomic condition with arteriosclerosis, vasculitis, loss of elasticity or fibromuscular dysplasia [11–14]. Of note, some authors suggest that both causes coexist in the same patient: the etiology of carotid kinking could have a congenital basis and may become exaggerated with aging of the artery [15]. In particular, kinking might be congenital, decrease or disappear during somatic growth, and then reappear with physiological changes in the elderly. To further elaborate on this topic, the aim of this study was to evaluate the prevalence of internal carotid coiling and/or kinking (ICCK) across age categories.

## Methods

From September 2015 to May 2019, consecutive adult patients underwent echo-color Doppler (ECD) examination at the angiology unit of the A.O.U. Policlinico “G. Rodolico-San Marco” in Catania, Italy due to multiple indications. This cohort was enriched with examinations from children of pre-scholar and scholar age obtained as part of a screening program. When participants had multiple examinations, the baseline study was considered. All subjects (or their parents if underage) gave their informed consent for recording and storing of ECD images and their use for this observational study.

**Fig. 1:**
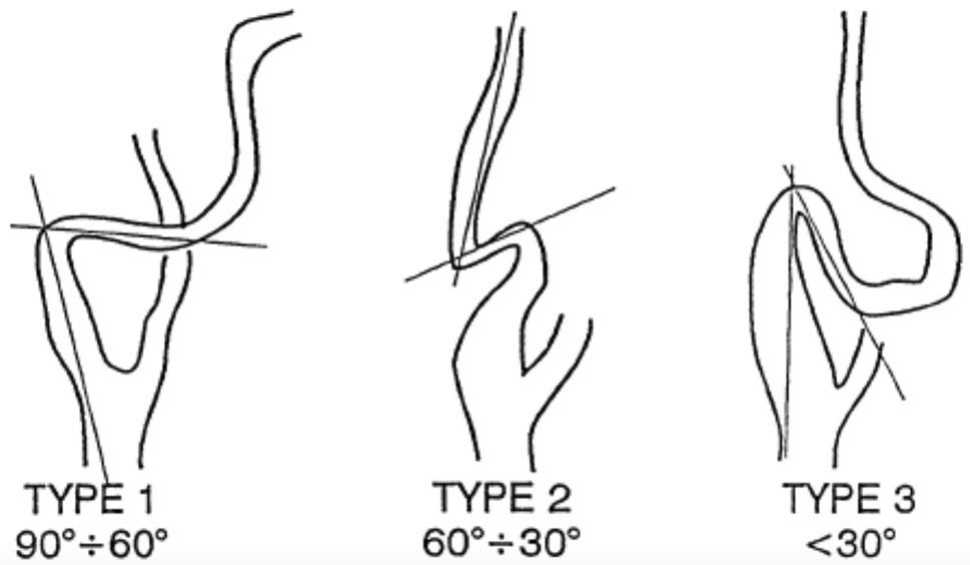
Metz’s classification of carotid kinking [8]. Two lines drawn along the axis of the internal carotid artery show an angle of curvature of the vessel less than 90° (type 1) or 60° (type 2) or 30° (type 3)

The EICA morphology of study participants was evaluated and subjects with ICCK were identified. Coiling was defined as an artery tract that describes a complete rotation for 360°. Kinking was defined as a morphological anomaly of the artery with an acute angulation (not greater than 90°). Kinking curves were classified according to the Metz classification according to the severity of the angle [8] (Fig. 1): Metz 1 or mild kinking (< 90°), Metz 2 or moderate kinking (< 60°) and Metz 3 or severe kinking (< 30°). Maximal velocity at the coiling or kinking level was recorded. The ratio between maximal velocity recorded at the kinking or coiling level and two centimeters proximal to the carotid bulb was defined as peak systolic velocity kinking ratio (PSVKR).

Data are reported as mean ± SD for continuous variables and as frequencies and percentages for categorical variables. Comparisons of variables was performed with the student t test (continuous variables) or chi-square test (categorical variables). Data analysis was performed with the Statistical Package for Social Sciences (SPSS) version 22.

## Results

During the observation period, ECD was performed in 2,856 subjects with age ranging between 0 and 96 years, including 1,632 males (57.1%) and 1,224 females (42.9%) (Table 1). ICCK were identified in 284 (9.9%) study participants, of which 138 were males (48.6%) and 146 were females (51.4%, p = 0.002). In patients with ICCK, 74 (26.1%) were bilateral, 99 (34.9%) were detected only in the right EICA and 111 (39.1%) were detected only in the left EICA. There was no difference in the mean age of subjects with and without ICCK (p = 0.88). However, the prevalence of ICCK was significantly different across decades (p < 0.001) and distributed in a bimodal fashion (e.g., with higher proportions in subjects ≤ 20 years old and > 60 years old; Table 2).

**Table 1:** Key characteristics of subjects with or without coiling/kinking of extracranial internal carotid arteries

Variables	All N = 2856	ICCK N = 284	No ICCK N = 2572	p value
Age, mean ± SD	58 ± 22	58 ± 26	58 ± 22	0.88
Males, n (%)	1632 (57.1%)	138 (48.6%)	1494 (58.1%)	0.002
Females, n (%)	1224 (42.9%)	146 (51.4%)	1078 (41.9%)	
EICA				
Bilateral, n (%)	–	74 (26.1%)	–	–
Right, n (%)	–	99 (34.9%)	–	–
Left, n (%)	–	111 (39.1%)	–	–

**Table 2:** Prevalence of ICCK stratified by age decades

Age groups (years)	Patients undergoing ECD of EICA (2856) (n)	Patients with ICCK (284) (n)	Prevalence, % (ratio)	
0–10	208	39	18.8	15.2% (57/374)
11–20	166	18	10.8	
21–30	55	2	3.6	4.4% (5/113)
31–40	58	3	5.2	
41–50	228	18	7.9	5.3% (36/682)
51–60	454	18	4	
61–70	777	59	7.6	11% (186/1,687)
71–80	680	90	13.2	
> 80	230	37	16.1	

**Fig. 2:**
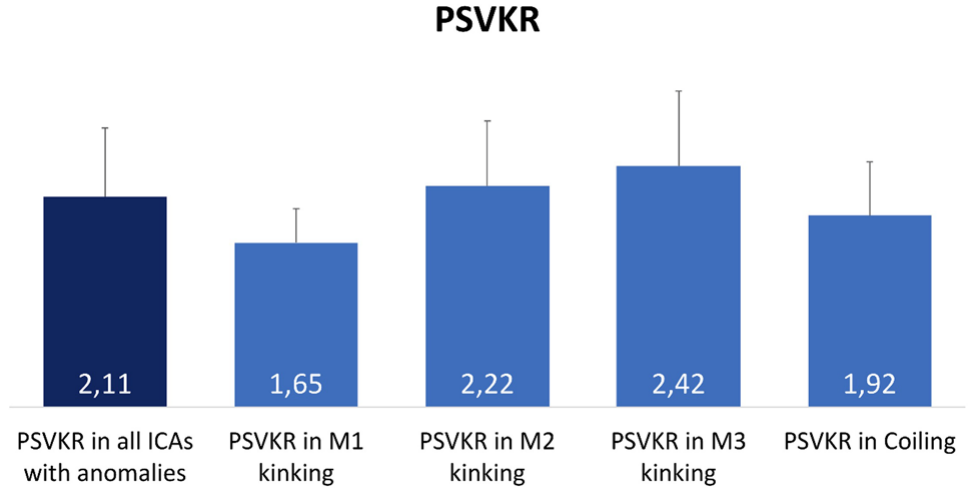
Peak systolic velocity kinking ratio (PSVKR) between the kink level and the common carotid artery, related to each grade of ICA anomalies. Blue bars indicate mean values. Error bars indicate standard deviations

Of a total of 357 EICAs affected by ICCK, coiling was detected in 10.1%, kinking with Metz grade 1 in 25.8%, kinking with Metz grade 2 in 30.8% and kinking with Metz grade 3 in 33.3%. (Table 3). The global mean value ± SD of PSVKR among all EICAs with morphological anomalies was 2.11 ± 0.69 (Fig. 2). The PSVKR ratio was numerically lower in arteries with coiling (mean ± SD 1.92 ± 0.54) and in those with grade 1 kinking (mean value ± SD were 1.65 ± 0.34), while it was higher in EICAs with more severe kinking (2.42 ± 0.75 in the Metz 3 group, 2.22 ± 0.65 in the Metz 2 group).

**Table 3:** Severity of morphological anomalies (kinking and coiling) of EICA, according to Metz’s classification

Age groups (years)	M1 (n)	M2 (n)	M3 (n)	Coil (n)	EICA with anomalies (n)
0–10	8	15	20	13	56
11–20	3	7	13	2	25
21–30	0	2	0	0	2
31–40	1	0	2	0	3
41–50	12	3	3	2	20
51–60	5	5	11	1	22
61–70	25	25	21	4	75
71–80	27	35	41	6	109
> 80	11	18	8	8	45
Total, all EICA with anomalies, n (%)	92 (25.77%)	110 (30.81%)	119 (33.33%)	36 (10.08%)	357 (100%)

## Discussion

The prevalence of EICA dolichoarteriopathies has been inconsistently reported in the literature, ranging from 2 to 43% [1, 2, 16–20]. Notably, in most of these studies, young subjects were under-represented and some studies also considered tortuosity among EICA abnormalities. In our study, including a mixed population of 2856 adults and children with focus on both kinking and coiling, the prevalence of EICA abnormalities was 9.9%.

Previous studies assessed the ratio of blood flow velocity (expressed as peak systolic velocity) in EICA and the common carotid artery using Doppler sonography [21], but data were obtained in healthy subjects or patients with EICA atherosclerotic narrowing (e.g., at variance with our study including subjects with kinking). Beigelman et al. performed a functional evaluation of EICA kinking by ECD and no differences were found between flow velocity (systolic and diastolic) measured at the level of kinking compared to the normal segment of the vessel [22].

We assessed the ratio between maximal velocity recorded at the kinking or coiling level and two centimeters proximal to the carotid bulb and named it peak systolic velocity kinking ratio (PSVKR). At variance with other metrics, PSVKR is a ratio between carotid tracts with and without anatomical anomalies, rather than between a stenotic and a non-stenotic segment. Peak systolic velocity and velocity ratio are basilar parameters in the evaluation of EICAs stenoses. According to the 2012 guidelines on diagnosis of vascular diseases and ultrasound investigations [23], a ratio of peak systolic velocity between two different arterial (carotid) segments of < 1.5 is considered physiological or indirect sign of a stenosis < 50%; a ratio of > 3.2 predicts a stenosis > 60%, while a ratio of > 3.3 predicts a stenosis > 70%. These guidelines do not indicate the peak systolic velocity ratio as an element for the evaluation of kinking-related stenosis, hence we adapted the definition for the purposes of our study.

Regarding the distribution of ICCK across age groups, we found an increasing prevalence in the older age groups (over 50–60 years), which is consistent with the literature [24]. However, to the best of our knowledge, previous studies did not include a pediatric population. This is necessary to support the hypothesis of dynamic changes of EICA dolichoarteriopathies at different ages, if any. In our population, 15.3% of subject under 20 years and 5.2% of subjects of 40 to 60 years old had kinking, supporting the hypothesis of a progressive reduction of kinking prevalence with growth. Due to the design of the study, we do not have follow-up data that substantiate this hypothesis with paired measurements, which warrants a cautious interpretation. Our data show peaks of prevalence in ICCK at the ends in younger and older patients (e.g., < 21 years and > 60 years), and a lower prevalence in subjects between 21 and 60 years (Table 2).

**Fig. 3:**
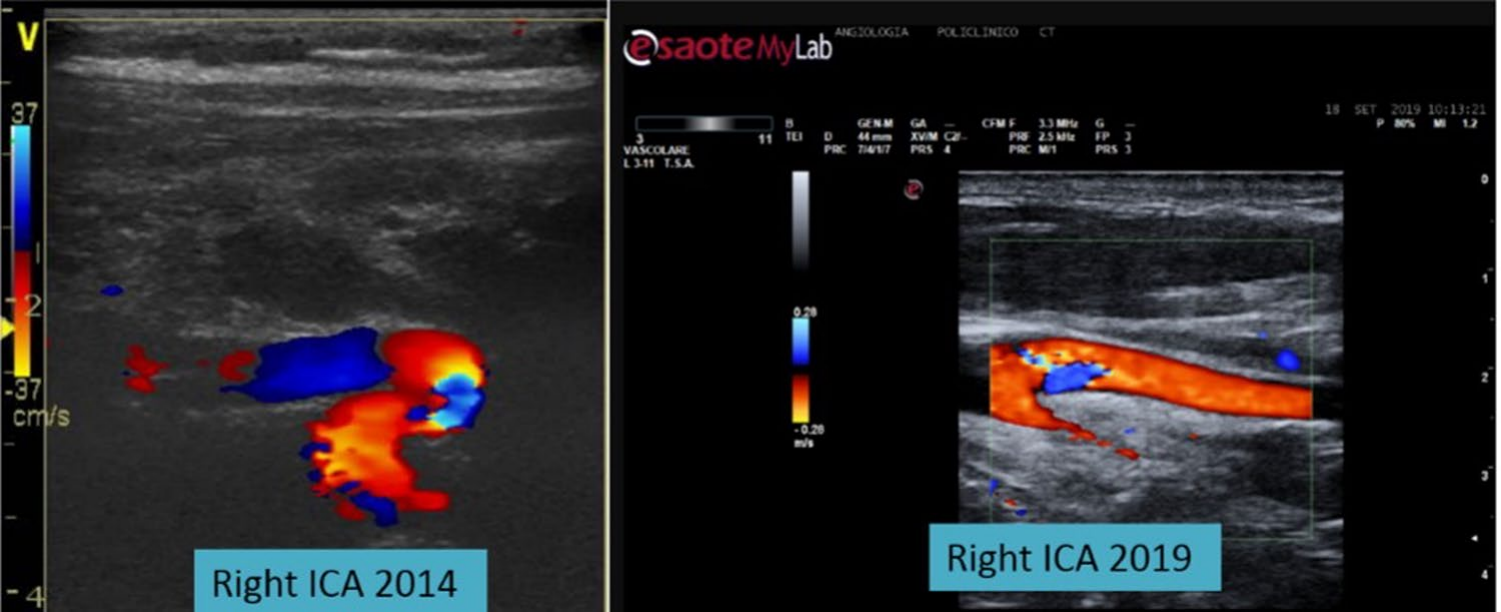
The disappearance of the internal carotid artery (ICA) kinking in a child five years after the first echo-color Doppler (ECD) study. This figure shows the image of ECD study of the right internal carotid artery (ICA) of a child in two different times in his life. **a** At the age of 11 years old (in 2014), he presented a morphological anomaly of right ICA with an angulation < 90°, i.e. a kinking. **b** Five years later (in 2019, when he was 16 years old), the ECD study of the same subject’s right ICA showed a total disappearance of the dolichoarteriopathy

**Fig. 4:**
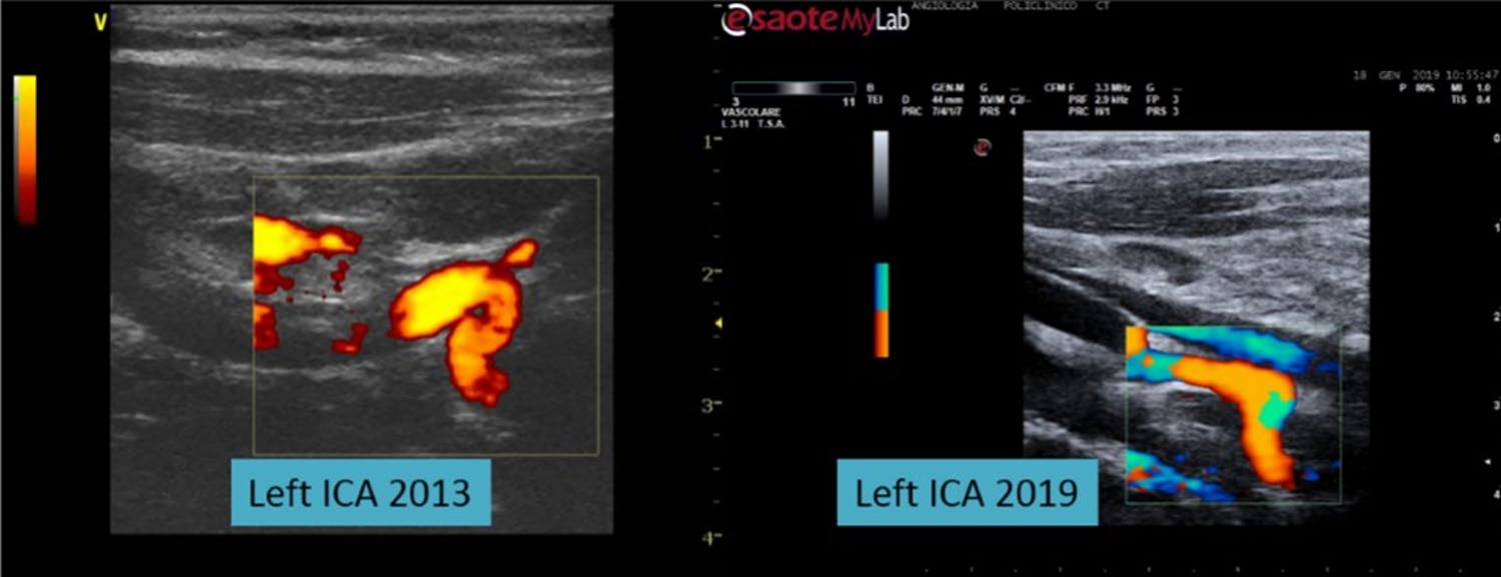
The reduction of the internal carotid artery (ICA) kinking to a simple tortuosity in a child six years after the first echo-color Doppler (ECD) study. This figure shows the image of ECD study of the left internal carotid artery (ICA) of another child in two different times in his life. **a** At the age of 9 years old (in 2013), he presented a kinking of the left ICA. **b** Six years later (in 2019, when he was 15 years old), the ECD study of the same subject’s left ICA showed a reduction of his anatomical anomaly to a simple tortuosity with an angulation > 90°

Similar to Morris et al. who suggest that EICAs anomalies decrease with age [25], we support the notion of ICCK intended as a sporadic congenital condition that with increasing of age and body growth diminishes and/or disappears because of the stretching of the aorta and supra-aortic trunks. To reinforce this idea we show in Figs. 3 and 4 anecdotal images of two children that had been undergone a ECD study of EICAs, documenting kinking, and a new ECD study a few years later. comparing the images of the same patients’ EICAs at two different times in their life highlight the reduction of the morphological carotid anomaly with growth. Conversely, in the elderly, ICCK may manifest because of senile crushing (e.g., osteoporotic vertebral compression) of the cervical spine and the neck and/or vascular remodeling secondary to atherosclerosis or hypertension or aortic arch’s elongation [12–14].

## Limitations

This is a single-center and hypothesis-generating descriptive study, which cannot draw conclusions about changes in tortuosity/kinking over time. Absence of ECD follow-up of the EICA anatomy among subjects with detected anomalies is another caveat. Hence, our study must be interpreted as a report of the prevalence of the EICAs anomalies in a large population spanning from 0 to 96 years. We did not include the evaluation of simple tortuosities (e.g., curves with an angle greater than 90°) because according to the literature they do not carry remarkable hemodynamic consequences (e.g., flow acceleration), whilst coiling and kinking cause hemodynamic abnormalities and are the most clinically relevant carotid anomalies [6–8].

## Conclusions

We determined the prevalence of ICCK in a large sample and its distribution in age groups. Our data support the hypothesis that kinking of EICA could be a physiological anatomic anomaly that improves over time but increases its prevalence in the elderly, following a bimodal distribution. Studies with long-term longitudinal follow-up are required to support this hypothesis. Consensus on criteria for defining pathological kinking and distinguish it from physiological kinking are warranted.

## Data Availability

All data and materials (taken from the department database) support published claims.

## Abbreviations

ECD: Echo-color Doppler
EICA: Extracranial internal carotid arteries
ICCK: Internal carotid coiling or kinking
SD: Standard deviation
